# Finding new grain forms in three dimensions

**DOI:** 10.1038/s41598-018-37279-y

**Published:** 2019-01-31

**Authors:** Hao Wang, Weihua Xue, Minnan Feng, Guoquan Liu

**Affiliations:** 1 School of Materials Science and Engineering, University of Science and Technology Beijing, Beijing, 100083 People’s Republic of China; 2 School of Materials Science and Engineering, Liaoning Technical University, Fuxin, 123099 People’s Republic of China; 3 Collaborative Innovation Center of Steel Technology, University of Science and Technology Beijing, Beijing, 100083 People’s Republic of China

## Abstract

Topological grain forms in three dimensions are studied experimentally and by large-scale Potts model Monte Carlo simulation. Some new band-faced grain forms are firstly observed among 16,254 pure iron grains, yet none of them is found among 28,049 Monte Carlo simulation grains, which indicate that there is shorter residence times for band-faced grain forms in three dimensions. The combined curvature/topology analysis suggests a possible efficient way of topological transitions of grain forms which is different from the known transition paths.

## Introduction

Cellular structures are very common in nature, for example, polycrystals in materials, foams in physics, biological tissues in biology^1^. Although differences are clear among different cellular systems resulting from different formation or evolution processes, they shares many features in common^2,3^ since they all belong to space-filling aggregates. Cellular networks are implicated in many, varied material properties at many different length scales, including lifetime properties such as fracture toughness, and functional properties such as electrical conductivity^4^. The intensive analysis on the characteristics of grain/bubble/cells forms is crucial for understanding of the cellular structures with an ultimate goal of preparing arrangements of grains and boundaries suitable for a given application.

Grain forms consist of the information of number of faces, edges per face and face arrangements, i.e., the refined/detailed grain topology. The topological viewpoint of grain growth describes the process as the decomposition of grains through decreasing numbers of faces toward the end state of a tetrahedral grain that disappears at a quadruple point^2^. The study on the paths of evolution of grain forms during grain growth is urgently need for describing the mechanism of grain growth from topological viewpoint. Nowadays, there are some important methods to describe the topological grain forms. E.A. Lazar *et al*.^3^ introduced Weinberg vectors^5^ and p vectors^6^ to describe the grain forms and investigated the topological difference between Poisson-Voronoi and grain growth microstructures. This study focuses on the relative stable grain forms (no two faces share more than one edge), yet grain forms containing “band-faces” (two faces share more than one edge) are not considered in^3^. Due to the fact that real grains have been observed with non-convex geometries including band-faces, T. Keller *et al*.^2^ and B.R. Patterson *et al*.^7^ considered both of the above two kinds of grain forms in their analysis respectively. T. Keller *et al*.^2^ studied the dispersion in the distribution of edges per face for each grain form in different cellular structures. B.R. Patterson *et al*.^7^ used Schlegel diagrams^8^ to study the paths of evolution of topological grain forms and propose some possible forms for grains with 4–9 faces. However, some of these grain forms are neither found in experiment nor in simulation.

W. Xue *et al*.^9^ developed a matrix description of grain’s refined topology and generated more possible grain forms than before by this new method. Since some topological forms for grains with 4–9 faces generated by either B.R. Patterson *et al*. or W. Xue *et al*. have not been found in experiment and simulation, verification of these forms and analysis on their evolution path is urgently needed. In this work, by using the largest experimental grain dataset to date^10^ and a much bigger Potts model Monte Carlo simulation grain dataset^11^, we verified some new topological grain forms and analyzed their possible evolution path in detail. The results also show clearly the difference between the experiment and simulation.

## Methods

Serial sectioning experiment was employed to reconstruct 3D microstructure of pure iron (99.9%pure). The specimen was sectioned from the center of a 30 mm diameter forged bar, and then annealed at 880 °C for 3 h, providing a completely recrystallized grain microstructure with a mean equivalent-area circle diameter of 32 mm. The reconstruction, visualization and quantification of pure iron microstructure was done in a scale of 1090 × 1730 × 540 μm^3^ on the basis of 300 serial 2D metallographic pictures. The detailed procedure of the 3D reconstruction method was seen in^12^. A total of 16,254 full 3D pure iron grains were used for topological analysis.

Besides the above natural grain growth microstructure, microstructures from simulations of normal grain growth using Monte Carlo-Potts model, phase-field method, interface-tracking method, etc. are normally used to study the topological properties of space-filling cellular networks. In this work, Monte Carlo-Potts model was used to simulate 3D normal grain growth in a large scale of 400^3^, which was described in detail previously^13^. We took steady-state grain-growth structure at 2500 Monte Carlo Step as research object, containing 28,049 Monte Carlo grains.

After the digital 3-D reconstructed pure iron microstructure and the Monte Carlo simulation structure were obtained, a program was developed^9^ to automatically get the number of grain faces *F*, edges *E*, vertices *V* and sketch the Schlegel diagram of each individual grain, which allows us to investigate the topological grain forms efficiently.

## Results and Discussions

**Figure 1:**
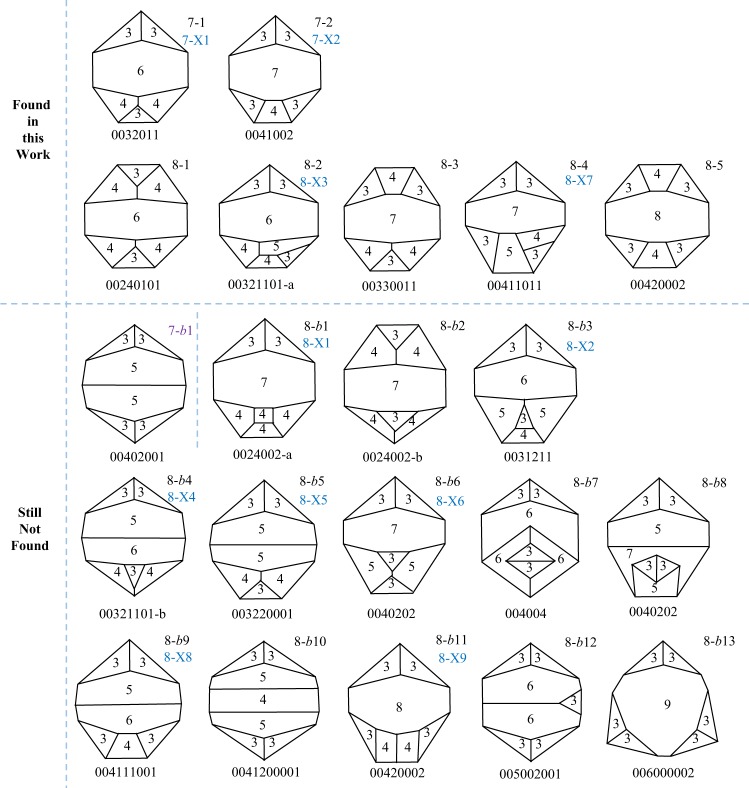
Schlegel diagrams of grains with band-faces for face class 7 and 8. Grain forms labeled black were predicted by present authors, while grain forms labeled blue were proposed in literature^6^. The seven forms above the horizontal dash line are first observed experimentally in this work.

Schlegel diagram of a given grain has been proven to be a good description of the topological grain forms. In the analysis of grain form evolution via Schlegel tree, Patterson *et al*.^7^ suggested 7 possible forms for 7-faced grains and 27 possible forms for 8-faced grains resulting from normal grain growth processes of face gain, loss and edge-switching events. Xue *et al*.^9^ recently has predicted that there are a total of 8 and 32 possible forms for 7-faced and 8-faced grains respectively, which completed the previous study in^7^. However, some of these forms had never been found experimentally or in simulation. Figure 1 shows all the undetected grain forms for 7- and 8-faced grains before this work. It is clear that all these forms belong to band-faced forms, i.e., grain forms with two faces sharing more than one edge.

We investigated the Schlegel diagrams of all grains in a pure iron microstructure (16,254 grains) and Monte Carlo simulated microstructure (28,049 grains). A total of 7 kinds of band-faced forms (28 grains included) was firstly found in pure iron microstructure, which consist of 553 7-faced grains and 606 8-faced grains in system. Thus, there was approximately 2.4% grains with band-faces among all the experimental data of 7-and 8-faced grains. This reinforces the importance of collecting statistically large datasets if one is to examine the topological forms of certain grain face class on a statistical basis. However, none of them was found in simulation. This somewhat shows the difference between the simulation and experiment structure.

The symmetry is one of the features of grain forms^3,7^. It is shown that some of the band-faced forms in Fig. 1 have good symmetry. Although the previous study^3^ suggest that highly symmetric grains are substantially common in the grain growth microstructure, band-faced grains with high symmetry are so rare in pure iron. Actually, these grain forms is observed to have a low algebraic connectivity besides high symmetry. The algebraic connectivity, λ_2_, is a new topological index to be used to describe the grain form characteristics^9^. It is influenced by not only the topological symmetry but also the edge distribution of grain faces. For highly symmetric topological forms, if they have a narrow edge distribution of faces, i.e., the number of edges of each face approximates the average edges per face, the values of λ_2_ is large. However, band-faced forms normally have a large-edged face and more 3-edged faces, i.e., a wide edge distribution of faces, resulting in a low algebraic connectivity. Thus, there is a relative low frequency of these forms due to the suggestion in^9^ that grain growth favors grain forms with a large algebraic connectivity.

Keller^2^ introduced the dispersion of a grain with *F* faces to study the characteristics of grain form,

1 $$\sigma ({\bf{p}})=\sqrt{\frac{1}{F}\sum _{i=1}^{n}{p}_{i}{(i-\bar{p})}^{2}}$$

2 $$\bar{p}=6-\frac{12}{F}$$

where *n* is the greatest number of edges on any face. The average number of edges per face $\bar{p}$ is known from Smith’s formula, Eq. (2) ^14^. Grains with large dispersion means that the number of edges of faces tend to deviate largely from $\bar{p}$. Since Monte Carlo simulation structure have the lowest dispersion, yet the pure iron structure has a much higher one, as reported in^10^, the large-dispersion band-faced forms is more likely to appear in pure iron rather than Monte Carlo structures. Real grain structures are affected by texture, second phase, processing history, etc., which induced instability effects in grain topology and possibly formed grain boundary with extremely large curvature, resulting in more complex grain forms with large dispersion. This serves as an explanation why we have found some band-faced grain forms in experiment rather than simulation.

**Figure 2:**
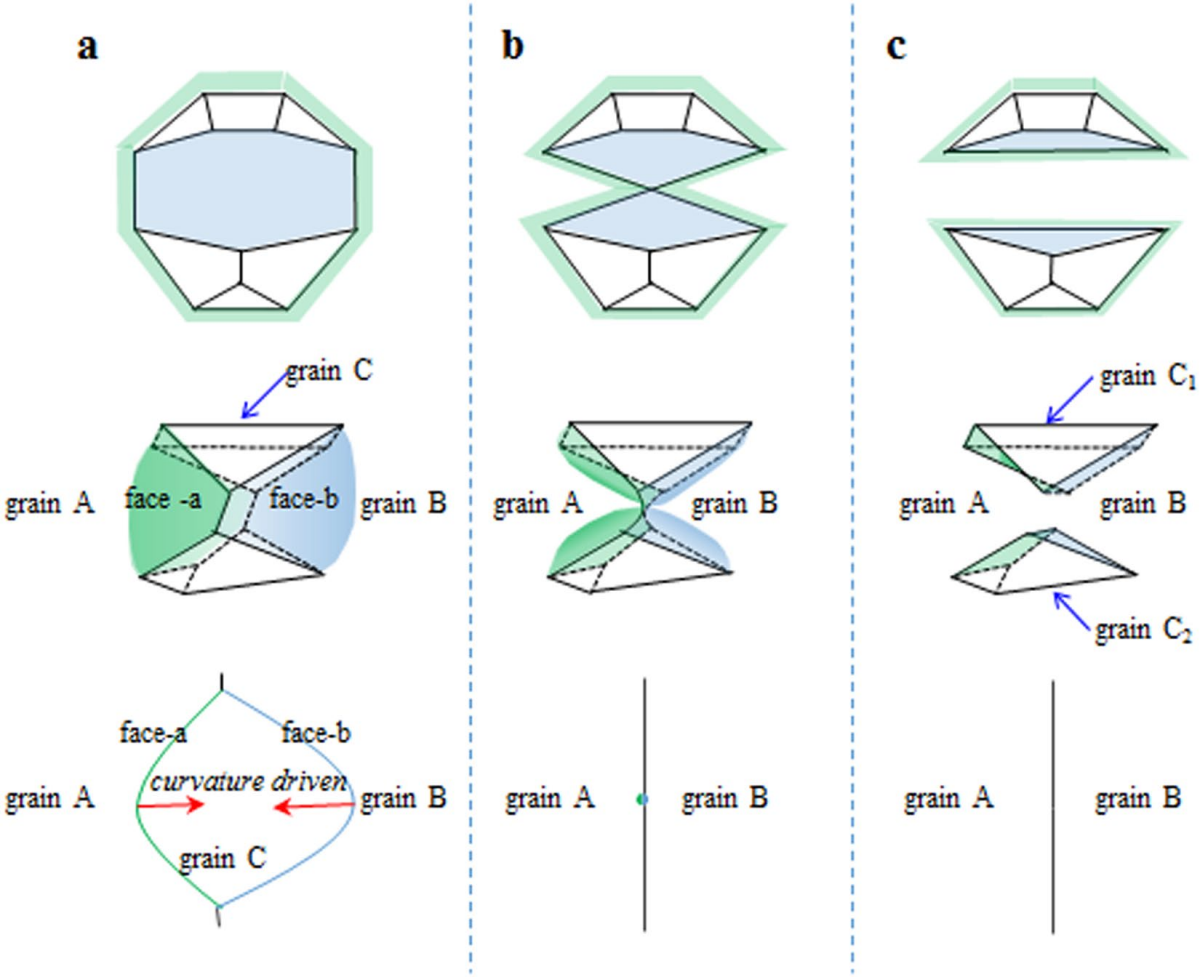
A possible path for the evolution of band-faced grains. The upper graphs are the Schlegel description of the evolution path, the middle ones are the corresponding 3D configuration, and the below ones are the cross section of the band-faces.

The evolution of the topological grain form is determined not only by the topological change between each grain and its neighboring grains, but also the boundary curvature that plays an important role in the kinetics of normal grain growth. According to the topology evolution analysis in^7^, there exist 3 kinds of evolution mechanism of grain forms including the loss, the addition of three-edged faces and edge-switching event. Here, from a combined curvature/topology view of grain growth, it seems there is another simple and fast way for evolution of the band-faced grains. Band-faces can only be found when the grain boundary is curved convex to a large extent as its neighboring grains are especially concave. Figure 2 suggest a new possible way of the evolution for grain 8-III with band-faces.

For the band-faced forms in Fig. 2a, face *a* and *b* share two common convex edges because they are all convex extremely. Under the curvature-driven mechanism, such convex boundary will lose its area rapidly in normal grain growth process. Figure 2b shows an instant time that the two common edges meet up together, resulting in the following separation of this grain into two parts, as shown in Fig. 2c. Such evolution path is more clearly demonstrated in a cross section of faces a, b and their common edges. Thus, the 8-faced grain forms are separated into two 5-faced forms, which decompose much faster than the sequential face reduction from 8 to 5. Due to the large curvature of the band-faces and such faster evolution path than usual, band-faced grains are very unstable and easily changing into other stable states, which account for the extreme low frequency of these band-faced forms in system.

Most of the topological forms will evolve into pentahedron and tetrahedron and then disappear in the system. The characteristic pairs of contiguous three-edged faces and their evolution is supposed to the reason why band-faced grains decompose rapidly from purely topological analysis^7^. The new evolution path of the band-faced grain forms present here should be another possible form evolution mechanism, which can supplement the previous result^7^ from a combined curvature/topology view.

## Conclusion

In summary, some theoretically predicted grain forms are firstly observed experimentally in a large grain dataset of pure iron grains, yet none of them are found in Monte Carlo simulation. These band-faced grain forms have low algebraic connectivity, large dispersions and are unstable in normal grain growth. A combined curvature/topology analysis suggests that the most possible evolution path for band-faced grains is to be broken into two new lower-faced grains at the common edges shared by two faces in one individual grain, which is different to the known paths of uphill, downhill and horizontal topological transitions, resulting in shorter residence times of these band-faced forms before transitioning to lower classes.
